# Food Difficulties in Infancy and ASD: A Literature Review

**DOI:** 10.3390/children10010084

**Published:** 2022-12-31

**Authors:** Marios Vasilakis, Konstantinos Polychronis, Eleni Panagouli, Efthalia Tzila, Alexia Papageorgiou, Loretta Thomaidou, Theodora Psaltopoulou, Maria Tsolia, Theodoros N. Sergentanis, Artemis K. Tsitsika

**Affiliations:** 1 MSc Program “Strategies of Developmental and Adolescent Health”, School of Medicine, National and Kapodistrian University of Athens, 115 27 Athens, Greece; 2 Department of Clinical Therapeutics, “Alexandra” Hospital, School of Medicine, National and Kapodistrian University of Athens, 115 28 Athens, Greece; 3 Department of Public Health Policy, School of Public Health, University of West Attica, 115 21 Athens, Greece

**Keywords:** autism spectrum disorder, infants, food difficulties, early life, lactation

## Abstract

Purpose: The aim of this literature review is to investigate the potential association between specific food difficulties and autism spectrum disorder (ASD) during the first two years of life. Materials: The search was conducted in PubMed, Google Scholar, Embase and PsycInfo databases. Results: Twenty-one studies were synthesized (3763 infants and children). Difficulties during breastfeeding, breast milk refusal and avoidance of taking solids have been linked to ASD. Infants with ASD have been referred to as picky eaters. Problematic mealtime behaviour during infancy has also been associated with ASD. Conclusions: The present review highlights the association between food difficulties, including problematic mealtime behaviours, food selectivity, breastfeeding difficulties and food refusal during infancy and ASD early during life, including the first months.

## 1. Introduction

Autism Spectrum Disorder (ASD) is a neurodevelopmental disorder affecting the way an individual interacts socially and communicates. It is characterized by restrictive and repetitive patterns of behaviors, interests, or activities. According to the fifth edition of Diagnostic and Statistical Manual of Mental Disorders (DSM-5), feeding features are also displayed in the context of insistence on sameness and inflexible adherence to routines or ritualized patterns [1]. 

Feeding problems are a common situation for 25%–35% of typically developing children [2] and occur in up to 80% of those with developmental delay [3]. It has been supported that individuals with ASD are five times more likely to display food difficulties [4,5,6]. Atypical eating behaviors such as food selectivity, food refusal, mealtime behaviors, unusual and ritualistic eating patterns have been examined in children with ASD [7]. 

The purpose of the present literature review was to investigate whether food difficulties during the first two years of life might be associated with ASD; to this end, evidence from cohort, case-control, cross-sectional studies, case series and case reports was synthesized. 

## 2. Materials and Methods

### 2.1. Study Design

The search was performed in the following databases: PubMed, Google Scholar, Embase and PsycInfo. Last search was performed on October 19, 2020. The algorithm used was the following: (“food difficulties” OR “feeding difficulties” OR “feeding difficulty” OR “food selectivity” OR “avoidant restrictive food” OR “food avoidance” OR “mealtime behavior problems” OR “food choice” OR “dietary patterns” OR “severe feeding problems” OR “atypical eating problems” OR “selective eating” OR “restrictive eating” OR “food refusal” OR “food phobia” OR “picky eating” OR “feeding challenges” OR “sensory food aversion” OR “food intolerance” OR “nutrient intake” OR “food sensitivity” OR “nutritional deficiencies” OR “nutritional intake” OR “nutritional status” OR “eating habits” OR breastfeeding OR “breast feeding”) AND (infancy OR infant OR infants OR toddler OR toddlers OR “early infancy” OR child OR children OR childhood OR kids OR kid) AND (ASD OR “autism spectrum disorder” OR autism OR autistic OR autistics). A snowball technique was conducted in order to search for relevant references of eligible studies and reviews.

### 2.2. Inclusion Criteria

Studies that examined the association between food difficulties in infancy (0–2 years old) with an ASD diagnosis were considered eligible for this literature review. Problematic mealtime behaviors, breastfeeding difficulties, food refusal and food selectivity (food preparation in a special way, avoidance or crave of certain foods, favorite food textures and picky eating) were considered food difficulties and they could have been reported by health care professionals through mother-infant observation, assessment of early history, health records, semi-structured interviews with parents/caregivers, standardized tests or questionnaires. All these behaviors must have been observed until two years old. Concerning the study design, cohort studies, case control, cross sectional, case reports and case series were selected; there were no gender, language or other demographic restrictions. 

Studies that fell in the following criteria were excluded: (1) Children older than two years old (2) infants or children with anatomical, oral or other pathophysiological dysfunction, and (3) ASD with comorbidities. The selection of studies was performed by two authors (M.V., K.P.) working independently.

Some studies included an age range from 0–17 years old and were considered studies with age admixture; in those studies, relevant data were retrieved for food disturbances regarding the 0–2 year’s age group, according to the inclusion criteria.

### 2.3. Data Extraction and Analysis

To extract data from the eligible articles, a piloted data extraction form was used. Data were reviewed simultaneously and independently by two reviewers (M.V., K.P.). The data were extracted comprised: Name of first author and year of publication, region/country where the survey was conducted, study period, study design, sample size, description of sample, age range, ascertainment and/or association with ASD, statistical analysis and main findings about food difficulties. Data abstraction was performed by two authors (M.V., K.P.) working independently; any disagreement that occurred was discussed and resolved through reviewers and team consensus. 

### 2.4. Quality Assessment

Risk of bias assessment was performed by two authors of the search team (M.V., K.P.) who assessed and independently screened each potential eligible study during the screening process through Newcastle-Ottawa Scale for cross-sectional studies (Modesti, 2016) [8], cohort studies (Stang, 2010) [9] and case-control studies, as appropriate.

## 3. Results

### 3.1. Selection of Studies

A total of 21 studies (3763 infants and children) were included in this review. Between those studies, eight of them reported data from the USA [5,6,10,11,12,13,14,15]. Three studies presented data from Italy [4,7,16]. Four studies retrieved data from the United Kingdom [3,17,18,19]. Furthermore, two studies were performed in Canada [20,21], one in Denmark [22], India [23], France [24] and Sweden [2]. Among the studies, twelve were cross-sectional [2,4,5,6,7,11,12,13,14,16,18,20], five were cohort studies [15,17,19,22,24], two were case series [3,21], one was a case-control study [23] and one was a case report [10] (Table 1).

### 3.2. Breastfeeding Difficulties

Breastfeeding difficulties in early infancy are usually manifested by frequent breaks and are expressed as sucking, irritable eating, slow eating, drinking small quantities, gagging, choking or spitting up, inadequate satiation and earlier interruption of breastfeeding. According to one study [5], 57%–64% of parents of ASD children reported difficulties during breastfeeding (*p* < 0.01), a rate that was significantly greater than 15% of mothers of typically developing children. Similarly, in another study [19] children who developed later ASD or autistic traits had apparent breastfeeding difficulties at 6, 15 and 24 months. 

Another study comparing ASD with typical development (TD) and non-TD children at 15, 18, and 24 months, discovered that the ASD group exhibited a great increase in breastfeeding difficulty [6]. However, a study conducted in the USA [14] examining eating behaviors at the first three months of life did not show any significant finding in the domains of irritable eating (*p* = 0.39), requirement of frequent feedings (*p* = 0.39) and gagging or spitting up (*p* = 0.69). Another study from the USA, which referred to the duration of breastfeeding [11], after interpretation of semi-structured interviews, demonstrated that 26.3% of the ASD sample disrupted breastfeeding within the first three months. It was also reported that 47.3% were not satisfied (no satiation) after being breastfed [11]. 

### 3.3. Food Selectivity

Food selectivity, meaning food preparation in a special way, avoidance or craving of certain foods, favorite food textures and picky eating, is considered as the most common feeding disorder among ASD individuals, with prevalence rates between 46%–89% [4]. A cohort study detected that children with ASD at the age of two years were very choosy concerning food in a rate of 20% compared to the control group with rate 9.5% (odds ratio, OR = 2.45, 95% confidence interval, CI: 1.36–4.43) [17]. Another cohort study showed that infants with ASD were more frequently reported as picky eaters at 15 months (OR = 1.15) and at 24 months (OR = 1.96) [19]. A case report of a 28-month-old female infant with ASD, who developed food selectivity and refused all foods simultaneously at the age of 15 months, has also been published [10].

Food selectivity by type (e.g., eating vegetables) or by texture is another component, related to factors such as sensory sensitivities and preference of sameness [6]. A cross-sectional study [12] examined food difficulties in 349 toddlers. Of them 74 presented selectivity by type (62% of them were diagnosed with ASD), while 92 reported selectivity by texture (31% of them were diagnosed with ASD). Similarly, results from another study comparing children with ASD versus non-ASD, pointed out that the ASD group had a significantly higher probability to manifest selectivity by texture (*p* = 0.004) and selectivity by type (*p* = 0.036) [15]. A study conducted in the USA [14] also examined food selectivity by texture; the screening of records from the early history of 24 infants (of whom 16 with ASD) revealed that they have often developed favorite food textures (*p* < 0.001). In addition, picky eating seems to be the predominant eating behavior identified among the ASD population [13].

### 3.4. Mealtime Behaviors

Parents reported that their children with ASD had any behaviors during mealtime, including issues with feeding routine, overeating, tantrums, gagging and a different emotional reactivity. Such mealtime behaviors were of great concern, and they increased progressively as their infant grew up. In the study by Provost et al., 25% of parents whose children were later diagnosed with ASD had expressed concerns about the mealtime behaviors of their infants even from their first week, 37% during the first year and 50% during the first one or two years [14]. Gray H et al. suggested that the first signs of a different eating behavior were obvious from the first three months [5]. 

At the age of six months, a prospective longitudinal cohort study reported that ASD infants could not establish a feeding routine (OR = 1.77) while such difficulties had been continuing by the age of 24 months (OR = 1.20) (Bolton PF et al., 2012). As stated in a cohort study of Bolton [19], infants who were later diagnosed with autism indicated a significant increase in feeding difficulties and fads at 15 and 24 months, while behaviors of overeating were not always associated with ASD. Correspondingly, selected clinical charts of ASD presented food overstuffing (*p* = 0.001) at the age of 15 months [15]. 

According to a cross sectional study with a sample of 190 preschoolers (0–2 years old) who later received an ASD diagnosis, mealtime behaviors included only crying (*p* < 0.001) [2]. In line with the aforementioned study, in a case report of a 28 month old autistic female from the USA, apart from crying, the child also had tantrums and screams when other people ate, accompanied by behavioral problems such as gagging followed by emesis at the end of the meal [10]. 

As stated in a cohort study [24], infants with ASD (n = 13) exhibited different anticipation than those with typical development (n = 14) when the spoon approached their mouth during the mealtime situation (*p* = 0.008). Apart from that, Bryson S et al. reported the case of an autistic female that, at the age of 12 months, was eating only with mom’s tickles or her slow singing or with a bottle with extreme difficulties to soothe [21]. 

### 3.5. Food Refusal

Children with ASD exhibited high rates of food refusal, which was manifested by behaviors such as passivity at feed times, small amounts of foods or poor appetite [3]. According to data of a cohort study, parents reported that their infants, who later developed ASD, started to refuse breast milk (OR = 1.24) at 6 months while there was an avoidance of taking solids (OR = 1.20) [19]. According to Bolton et al., “ASD has been associated with a decrease in accepting to eat certain foods (OR = 0.91)” [19]. Through a chart review study from which 45% of ASD children and 54% of language delayed children were below 24 months, the prevalence at refusal to eat new foods was 10.3% in the ASD group versus 0% in children with language delay (*p* = 0.002) [15]. Difficulties in the introduction to new foods and consequently refusal to new foods were pointed out by Cornish as a difficult transition from smashed food to solid food among autistic children [18].

Case series pointed to the same direction. Problems with solid foods were reported in the ASD case of Keen DV et al. [3] where the infant refused to ingest them until two years of age, accompanied with overmuching of milk up to vomiting. Bryson et al. reported the case of a 12 month old child later diagnosed with ASD consistently refused all foods that were not smooth [21]. According to Cornish [18], all parents of ASD cases (n = 17) reported that their babies were eating everything but suddenly at 12 months a nutritional regression to eating nothing was noted, accompanied by loss of skills and delay in their development [18]. The same occurred in a case report by Barnhill et al., where an infant aged 15 months started to refuse all kinds of foods simultaneously and was later diagnosed with ASD [1]. 

### 3.6. Risk of Bias

Most of the studies were cross-sectional (n = 12). The score through Newcastle Ottawa Assessment Scale varied; in four studies, the quality score was low (score: 3–4), in three the score was moderate (score: 5–6) and in the remaining six, the score was high (score: 7–10). The main limitations pertained to the representativeness of the sample in conjunction with the small sample size which was not justified (n = 9). Additionally, another factor limiting the quality of eligible studies had to do with the fact that there was no description of the response rate or the characteristics among responders and non-respondents (n = 8). However, the score in cohort studies (n = 5) varied from fair to good quality, whereas the quality of the case-control study was moderate (score: 4). 

## 4. Discussion

The present literature review highlighted correlations between food difficulties and ASD among infants zero–two years old. The main food difficulties during infancy which might be present a connection with ASD diagnosis, include early breastfeeding issues, food selectivity, worrisome mealtime behaviors and food refusal. 

According to Provost et al., concerns about different eating behaviors might be reported by parents of ASD children even from the first week of life (25%), while they seem to increase gradually in the first year (37%) and between first and second year (50%), [14]. 

Parents often also experienced a situation of picky eating with their ASD children [6]. They also reported that feeding issues may be apparent during the first year of life [15] while concerns about their children arose at the age of 13 to 19 months [22]. In fact, this might often occur before an ASD diagnosis takes place between the second and third year of life [18].

This review indicated a crucial period from 6 months to 24 months regarding food difficulties in children with ASD. Seiverling et al. reported that the prevalence of refusal of eating new foods was 10.3% in the ASD group whose age was below 24 months versus 0% in those with language delay (no ASD diagnosis) (*p* = 0.002). Moreover, infants with ASD were not able to establish a feeding routine [19], while various studies pointed out the difficulty of the transition from smashed food to solid food, accompanied with excessive drinking of milk up to vomiting [3,18].

Moreover, ASD has been associated with a sudden and complete refusal of all foods at 15 months of age [10,21], predominantly regarding all foods that are not smooth [21], a pattern that was accompanied by loss of skills and delay in their development at 12 months [18]. At the same age, ASD has been linked to behaviors of overeating (OR = 0.78) [19], food overstuffing (*p* = 0.001) [15] and food selectivity (OR = 1.15) [19]. 

ASD has been associated with higher probability for food selectivity by texture (*p* = 0.004) and food selectivity by type (*p* = 0.036) [15], while Ashley et al. added sensory sensitivities and preference on sameness [6]. Food selectivity is a common characteristic in ASD children characterized by sensory aversions and a sense of disgust [25] while another study attributed this to a desire to maintain sameness and to resist to change [26]. Likewise, results from studies showed that avoidance of new foods and ritualistic eating behaviors are related to resistance to change [27] while limited food preferences have been associated with mealtime behavior problems [28] and tantrums [29].

Other studies have also highlighted that food difficulties during infancy may be an early sign of ASD [30]. While worrisome eating behaviors are common in the general children population, the prevalence in ASD children is higher [31] and varies from 51%–89% [32] while the rate of such behavioral feeding problems has been reported to be as high as 89% [33,34]. 

Limited food preferences or hypersensitivity to food texture or eating specific brands of food or pocketing without swallowing have been linked with ASD [13]. It is tempting to envisage that the identification of such behaviors in infancy would lead to early screening [11] as they may conceal ASD and a possibility of failure to thrive [2], signaling the need for appropriate early intervention [14,16].

The main limitation of the present review was that data concerning food difficulties and eating patterns in infancy were often derived retrospectively, namely many years after the ASD diagnosis, compromising the validity of results. Additionally, a control group was not present in all studies, for example case reports or small case series and other type of studies as well. Details about control and comparison groups are presented in Table 1. Finally, study quality was compromised in some of the included studies, as it was observed during the risk of bias assessment

Concerning the strengths of our study, a large sample, derived from many countries around the world, was synthesized in the present review.

## 5. Conclusions

In conclusion, the present literature review highlighted the correlations between food difficulties, problematic mealtime behaviors, breastfeeding difficulties, food refusal and food selectivity during infancy and ASD diagnosis. ASD-related food difficulties sometimes arose during the first months of life, preceding ASD diagnosis. Thus, the existence of consistent food difficulties could signal the need for further investigation, including an indication of ASD.

## Figures and Tables

**Table 1:** Description of included studies.

First Author (Year)	Region, Country	Study Period	Study Design	Sample Size	Age Range	Selection of Sample	Main Findings
[5]	Florida, USA	July 2017–March 2018	cross-sectional	41 (typically developing control from the study)	2–17 years old	Through the Center for Autism and Related Disabilities (CARD), other local centers for autism, clinics, and schools, parents were invited to participate in the study Parents completed a 48-question survey via Qualtrics online software.	69% of parents with ASD reported difficulties with breastfeeding which was significantly greater than 15% of mothers of typically developing children. 57–64% of parents with ASD aged 3–6 years reported difficulties with breastfeeding (*p* < 0.01).
[10]	USA	NR	case report	1 (no controls)	28 months	From a short-term outpatient nutritional and behavioral feeding intervention program.	Mother stated the child’s refuse to come nearby new foods. Expressed tantrums, cry, screaming when other people ate. When food entered her mouth, she noted gagging up to emesis. Denied all liquids than breast milk, only little water rarely. Drinking from straw but choking.
[7]	Rome, Italy	NR	cross-sectional	60 ASD children with a control group of 50 typically developing Italian children	20–44 months	From the Child Neuropsychiatry Unit of the Policlinico Umberto I, in Rome, Italy	Significant differences found between both ASD groups vs controls (*p* < 0.001) concerning food refusal.
[22]	Denmark	1996–2012	cohort	1313 (973 children with ASD and a control group of 300 children with intellectual disability)	At time of ASD diagnosis 8 years, at the end of follow up 11 years	By telephone interviews at the 12th week of pregnancy, 6 & 18 months. Identification of children through linkage of the cohort via the unique personal identification number with the Danish Psychiatric central Research Register	Mothers of ASD gave up on sole breastfeeding within the first 3 months (HR = 1.8–1.9). *7*% of the ASD group (n = 42), HR = 1.8 (95% CI)-(1.3,2.4). and 7.1% of the Child Autism group (n = 16), HR = 1.8 (1.1–3.1) have never breastfeeded. 14.8% of the ASD group have stopped breastfeeding at 1st month of age, (n = 89), HR =1.8 (1.4–2.3) and 23.6% (n-142) at 2–3 months. Only 5.8% have continued breastfeedin after 6 months of age (n = 35).
[4]	Italy	2009–2016	cross-sectional	163 (no controls)	20–71 months	From a tertiary care university hospital.	40.5% of ASD had at least one Gastro Intestinal symptom or Food Selectivity. Children with food selectivity showed more stereotyped behaviors (*p* = 0.003 Cohen’s d = 0.54) and ritualistic behaviors (*p* = 0.002, Cohen’s d =0.55).
[11]	USA	NR	cross-sectional	19 chlidren and 16 mothers (no controls)	5 to 6 years at the time of the interview, 18 months to 8 years—at the time of diagnosis	Through posters, flyers, an information table at ASD community events, and two community clinics that serve families with ASD.	According to the results of this study, 12 out of 19 children have initiated breastfeeding without difficulty and 14 have established breastfeeding with success. Among them, only 6 have breastfed beyond 6 months. Moreover more than half of the mothers (9/16) have described that their infants presented a dysregulated feeding pattern of vigorous sucking without stopping of their own volition. Authors have concluded that this pattern could be evaluated by clinicians in the general pediatric population and/or at-risk infant siblings of children with ASD.
[23]	India	May 2015–June 2016	case-control	30 children with ASD vs 30 typically developed children	2–6 years old	From a child guidance clinic of a tertiary care teaching hospital in India	Comparison of feeding practices of children with ASD vs Typically developing siblings: Exclusive breastfeeding up to 6 months: Children with ASD: (n = 13), 43.3%. Typically developing siblings: (n = 23), 76.7%. Comparison X2 = 5.625. Early introduction of top feeds: Children with ASD: (n = 17), 56.7%. Typically developing siblings: (n = 7), 23.3%. *p* = 0.0177.
[17]	Avon area, England	April 1991–December 1992	cohort	79 children with ASD vs 12901 controls	28 months	Data concerning feeding and food frequency were collected by questionnaires completed at 6, 15, 24, 38 and 54 months by caregivers in the Avon Longitudinal Study of Parents and Children	Slow feeding (1 month): Children with ASD: (n = 35) 47.3%, Controls: (n = 4.894) 40%, OR (95% CI)= 1.35 (0.85–2.14), *p* = 0.20. Slow feeding (6 months): Children with ASD: (n = 28) 40,6%, Controls: (n = 3497) 30.7%, OR (95% CI)= 1.66 (1.02–2.69), *p* = 0.04. Very difficult to feed (15 months): ASDs:(n = 6) 8.1%. Controls: (n = 374) 3.4% OR (CI 95%)= 2.71 (1.16–6.31) p 0.02. Very difficult to feed (24 months): ASDs: (n = 11) 15.5%. Controls: (n = 467) 4.5% OR (CI 95%)= 3.67 (1.91–7.05) *p* <0.01. Very choosy (15 months): ASDs: (n = 7) 9.5%. Controls: (n = 595) 5.4% OR (95% CI) = 1.92(0.87–4.21) *p* = 0.10. Very choosy (24 months): ASDs: (n = 14) 20%. Controls: (n = 979) 9.5% OR (95% CI) = 2.45 (1.36–4.43) *p* = 0.03.
[12]	USA	NR	cross-sectional	349 Autism (n = 26) Down syndrome (n = 21) Cerebral palsy (n = 44)	1 month-12 years old	Participants were collected from an interdisciplinary feeding program for the evaluation of feeding problems	A total of 26 children have been diagnosed with autism in the studied group. Among them 12% pesented food refusal, 62% selectivity by type, 31% selectivity by texture, 15% oral motor delay and 12% dysphagia. All the children with autism who presented food refusal had gastro-oesophageal reflux. The prevalence of food selectivity by type was found to be significantly higher for children with autism (χ2 = 27.49; *p* < 0.001).
[6]	USA	NR	cross-sectional	19	15–36 months	Those children were participants in a larger longitudinal infant sibling study that observes early development to better characterize early manifestations of ASD	ASD group exhibited greater increase and more rapidly in feeding difficulties over time compared with Non -Typical Development, High Risk-Typical Development, Low Risk-Typical Development with significantly more feeding difficulties by the age of 36months.
[3]	London, United Kingdom	NR	case series	7 (no controls)	Ages at diagnosis: case1 = 3.10 years old. case 4 = 3.8 years old. case 5 = 3.11 years old. case 7 = 5.4 years old (case 2.3.6 were rejected due to medical problems)	These seven children come from the author’s clinic population of approximately 350 children with autism, diagnosed following assessment in the child development services of two UK health districts	All 7 cases presented problems weaning to solids and some kind of abnormal food behavior. 2 cases (3 + 4) presented breastfeeding or bottle feeding problems. The presence of such persistent feeding problems should alert clinicians to the possibility of autsim.
[15]	Hudson Valley, New York, USA	1999–2005	chart review	ASD children (n = 78)/vs children with language difficulties (n = 85):	9–36 months	From University Center for Excellence in Developmental Disabilities which had sufficient information and documents coming from families who asked help there for either diagnostic evaluation or service coordination. were not children with primarily health, motor, or global developmental delays, appeared to be best candidates for the 2 groups (ASD and LD)	Food Selectivity by Texture: ASD 23.1% /LD 7.1% (chi square 8.31, *p* = 0.004), Food Selectivity by Type: ASD 24.4% /LD 11.8% (chi square 4.41, *p* =0.036), New Food Refusal: ASD 10.3% /LD 0.0% (chi square 9.17, *p* = 0.002), Food Overstuffing: ASD 14.1% /LD 3.5% (chi square 5.79, *p* = 0.001), Mean of four feeding problems for ASD group:0.72 (0.98) (0–4)/LD 0.22 (0.47) (0–2) t = 4.16 df = 161 *p* = 0.000). Significant main effect for gender was found with male children showing more feeding problems than female children (male: M = 0.59, SD = 0.89 and female M = 0.16 SD = 0.43). No significant main effect was found for age of first evaluation or neighborhood income and no significant Diagnostic Group X gender interaction effects appeared.
[13]	USA	NR	cross sectional	1112 (no controls)	1–17	Children that underwent a comprehensive diagnostic evaluation by a licensed PhD psychologist. (semi-structured diagnostic interview with the parents using the Checklist for Autism Spectrum Disorder, CASD)	The prevalence of picky eaters in the sample with autism was higher than in the sample in toddlers in general population.
[24]	France	NR	cohort	13 ASD and 14 typical devellopment	3–6 months	From family movies collected from psychologists in France who spoke French and an institution for people with autism in Flanders who spoke Dutch.	Infants with ASD had a deficit in mouth-opening anticipation while being fed in relation to typically development (TD) children. Although there is no difference in the number of attempts and the age, there was significant difference regarding the anticipation disorder (t(29) = −2.86; *p* = 0.008). The anticipation success depends on group (chi square (1) = 3.95; *p* = 0.047), meaning that fewer infants succeed in anticipating in the ASD group VS TD group.
[18]	South Derbyshire Health Authority	NR	cross sectional	17 (no controls)	42–117 months	identified by the Consultant Community Paediatrician	All parents of 17 cases (100%) reported a food refusal and also difficulty in introduction of new foods into their diet. They also stated that as babies they were eating everything and suddenly hardly did they eat anything. This occurred between 12 months and 3 years combined with loss of skills and other developmental delays.
[20]	Canada	1 January 2006–30 September 2006	cross sectional	48 children with ASD and their sibblings (n = 48)	3–12 years	Registered in one of four local rehabilitation centers, one tertiary pediatric hospital or one of two parent associations in Quebec, Canada.	It is important to screen children for eating difficulties with ASD as they had more eating problems and more mealtime behaviors than their typically developing siblings (F(1.45) = 23.24, *p* = 0.001). ASD children had a significant history of eating difficulties as infants in relation to their siblings t = 3.87, *p* < 0.001. No difference in duration of bottle feeding, breast feeding and use of pacifier between the two groups.
[14]	USA	NR	cross sectional	24 children with ASD vs 24 with typical development (TD)	3–6 years	Selected from a University Center for Excellence in Developmental Disabilities (UCEDD) which offers services.	47% of mothers who nursed their ASD child had breastfeeding difficulties while only 20% of mothers who nursed their TD child reported such difficulties (*p* = 0.34). As their child was growing up, concerns were increasing.
[16]	Italy	February 2001–July 2011	cross sectional	105 from which 57 cases idiopathic (54.3%) and 48 non-idiopathic (45.7%) with no significant differences between them.	0–51 months	Hospitalized at the Autism Centre of the Neurological Clinic of the University of Bologna.	Early ASD signs in the whole sample: 43.8% had feeding disorders. From them, selective eaters: 25.7%, scarce feeding: 11.4% and other feeding disorders: 23.8%. Feeding problems were more frequent (*p* = 0.031) in cases with delay (50%) and stagnation (53.3%) than in cases with regression (22.2%).
[2]	Sweden	NR	cross sectional	190 children with ASD and 161 children (comparison group)	0–2 years	from Child Health Centre (CHC)	Regulator problem (RP) in infancy was very common in children later diagnosed with ASD. The number of consultations for feeding was significant higher in the ASD group compared to the non ASD group U = 12745 (z = −3.38, *p* = 0.001). From 190 ASD children, the 105 parents of them filled out DISCO interview and there was correlation between sleeping problems (rs = 0.37, *p* < 0.001) and feeding problems (rs = 0.21, *p* = 0.033)
[19]	Bristol, England	April 1991-December 1992	cohort	86 (no controls)	0–30 months	Children in the Avon Longitudinal Study of Parents and Children (ALSPAC) with ASD identified either from community pediatric records or from the special educational needs databases for the region.	Children who exhibited later ASD or autistic traits, already exhibited developmental differences at 6 months including vision, hearing, feeding and bowel habit. There was a significant difference after Bonferrori adjustment (*p* < 0.00021) at 15 months old in feeding difficulties and fads.
[21]	Canada	NR	case series	9 (no controls)	6–36 months	The first nine children from a prospective study of infant siblings, recruited from three multidisciplinary autism diagnostic and treatment centers in Canada, including McMaster Children’s Hospital in Hamilton, the Hospital for Sick Children in Toronto, the IWK Health Centre in Halifax and from clinicians in the surrounding regions.	case 1/male: at 12 months refused food not smooth in consistency. Case 2/female: at 12 months limited food preferences. Case 3/female: at 12 months eats variety of foods but being fussy and with difficulties to soothe. Settlement with mom’s tickles or her slow singing or with a bottle. Case 4/male: not reported feeding. Case 5/male: at 10 months resists being fed. Case 6/female: not reported feeding. Case 7/male: not reported feeding. Case 8/male: no feeding problems at 12 months but 24 months limited food preferences (cheese, fruit, starches). Case 9/female: at 18 months strong food preferences—spits out meat and eats fish only if pureed).

HR: Hazard Ratio.

## Data Availability

Data available upon request.
